# *Lactobacillus paraplantarum* 11-1 Isolated from Rice Bran Pickles Activated Innate Immunity and Improved Survival in a Silkworm Bacterial Infection Model

**DOI:** 10.3389/fmicb.2017.00436

**Published:** 2017-03-20

**Authors:** Satoshi Nishida, Masaki Ishii, Yayoi Nishiyama, Shigeru Abe, Yasuo Ono, Kazuhisa Sekimizu

**Affiliations:** ^1^Genome Pharmaceuticals Institute Co. Ltd.Tokyo, Japan; ^2^Laboratory of Microbiology, Graduate School of Pharmaceutical Sciences, The University of TokyoTokyo, Japan; ^3^Department of Microbiology and Immunology, Teikyo University School of MedicineTokyo, Japan; ^4^Teikyo University Institute of Medical MycologyTokyo, Japan

**Keywords:** Lactic acid bacteria, *Lactobacillus* sp., silkworm, innate immunity, infection, *Pseudomonas aeruginosa*

## Abstract

Lactic acid bacteria (LAB) have high immune system-stimulating activity and are considered beneficial for human health as probiotics in the gut. The innate immune system is highly conserved between mammals and insects. Microbe-associated molecular patterns (e.g., peptidoglycan and β-glucan) induce cytokine maturation, which, in silkworm larvae, leads to muscle contraction. The purpose of this study is to find a novel probiotic by using silkworm muscle contraction assay. In the present study, we isolated LAB derived from rice bran pickles. We selected highly active LAB to activate the innate immune system of the silkworm, which was assayed based on silkworm muscle contraction. Of various LAB, *L. paraplantarum* 11-1 strongly stimulated innate immunity in the silkworm, leading to stronger silkworm contraction than a dairy-based LAB. Silkworms fed a diet containing *L. paraplantarum* 11-1 exhibited tolerance against the pathogenicity of *Pseudomonas aeruginosa*. These findings suggest that *L. paraplantarum* 11-1 could be a useful probiotic for activating innate immunity.

## Introduction

Innate immunity is highly conserved between invertebrates and vertebrates. In mammals, dendritic cells and macrophages produce cytokines in response to microbial pathogens (Janeway and Medzhitov, 2002), whereas in insects, hemocytes and fat bodies recognize microbial pathogens and induce an anti-microbial response (Brennan and Anderson, 2004). Especially in silkworms, immune cells produce reactive oxygen species to activate proteases, resulting in cytokine release. Our group discovered an active cytokine, paralytic peptide, that induces muscle contraction in silkworms (Ishii et al., 2008). We used silkworm muscle specimens to screen for innate immunity-activating substances and found that lactic acid bacteria (LAB) strongly induce silkworm muscle contraction (Dhital et al., 2011; Fujiyuki et al., 2012; Nishida et al., 2016). This screening method has several advantages compared with conventional screening using mammalian innate immune cells such as macrophages. First, the silkworm does not respond to lipopolysaccharides, which often produces a false-positive response in mammalian macrophages. Second, insect whole body assays reflect the absorption, distribution, metabolism, excretion, and toxicity factors that govern the therapeutic effects of medicines. Therefore, substances with less effective pharmacokinetics/pharmacodynamics and/or are toxic in the silkworm muscle contraction assay would be excluded by these tests.

LAB are traditionally used for fermenting foods, dairy products, and probiotics. LAB are Gram-positive, catalase-negative, form no spores, and are immotile. Foods fermented with LAB could be beneficial for human health by activating innate immunity (Ichikawa et al., 2012; Kawashima et al., 2013). In our previous report, we isolated a dairy-based LAB to activate the innate immune system in the silkworm (Nishida et al., 2016). LAB isolated from dairy products have been characterized well, whereas LAB from fermented pickles have not. The purpose of this study is to find a novel probiotic to activate the innate immune system in the silkworm by screening LAB from non-dairy products, such as fermented pickles.

In this work, we isolated LAB from rice bran pickles and Korean pickles (kimchi). We evaluated the innate-immunity stimulating activity of LAB in silkworms. We selected a highly active LAB based on the results of the silkworm muscle contraction assay. Isolated *L. paraplantarum* 11-1 exhibited high activity in the silkworm contraction assay. Silkworms that ingested an artificial diet containing *L. paraplantarum* 11-1 exhibited tolerance against the pathogenicity of *Pseudomonas aeruginosa*. To the best of our knowledge, this is the first report of a probiotic effect of *L. paraplantarum* against *P. aeruginosa* infection. This LAB might be valuable as a probiotic for activating innate immunity. The infection model used in this study has the potential to be used to study a novel probiotic against *P. aeruginosae*.

## Materials and methods

### Materials

Gifu Anaerobic Medium (GAM) broth and GAM agar were purchased from Nissui (Tokyo, Japan). MRS broth and MRS agar were purchased from Becton Dickinson (Franklin Lakes, NJ, USA). CaCO_3_-MRS agar was prepared by adding CaCO_3_ (final concentration: 1%, Wako, Osaka, Japan) to the MRS agar after autoclaving. An AnaeroPak (Mitsubishi Gas Chemicals, Tokyo, Japan) was used for anaerobic culturing on agar plates. Saline was prepared as 0.9% NaCl (Wako, Osaka, Japan). Lysogeny broth (LB) medium was prepared with 1% bacto tryptone (Becton Dickinson, Franklin Lakes, NJ, USA), 0.5% bacto yeast extract (Becton Dickinson, Franklin Lakes, NJ, USA), and 1% NaCl (Wako, Osaka, Japan). An LB agar plate was prepared with LB medium containing 1.5% (w/v) agar (Nacalai Tesque, Kyoto, Japan).

### DNA sequencing

Fragments containing 16S rDNA were amplified with polymerase chain reaction using KOD FX Neo (Toyobo, Tokyo, Japan) with primers 9F and 1541R (Hashimoto et al., 2007). The DNA sequences were determined with direct sequencing, BigDye Terminator v3.1 Cycle Sequencing Kit and ABI PRISM 3100 Genetic Analyzer (ThermoFisher Applied Biosystems, Foster City, CA, USA). Sequences were analyzed with the NCBI BLASTN 2.2.27+ (Zhang et al., 2000), 16S ribosomal RNA sequences database (Bacteria and Archaea 7545 sequences). DNA sequences are currently in preparation for submission to the GenBank.

### Characterization of LAB

Fluid from the pickles was spread on MRS agar. After incubation at 30°C for 2 days, white colonies appeared on each plate. Isolated bacteria were Gram-stained with Gram-color (Merck, Kenilworth, NJ, USA). For scanning electron microscopy (SEM), bacterial cells were pre-fixed with 2.5% glutaraldehyde in 0.1 M cacodylate buffer (pH 7.2), post-fixed with 1% osmium tetroxide in the same buffer, and freeze-dried in *t*-butyl alcohol. The sample was examined with a field-emission SEM (JSM- 7500F, JEOL, Japan). The bacterial colony was suspended in 3% H_2_O_2_ for a catalase test. *Staphylococcus aureus* RN4220 and *Escherichia coli* JM109 were used as controls. Other identification kits, Api Zym and Api 50 CHL, were purchased from bioMérieux (Marcy l'Etoile, France), and the data were analyzed using the Api web v5.1 database (bioMérieux, Marcy l'Etoile, France).

### Silkworm muscle contraction assay

Silkworm muscle contraction was measured as previously reported (Ishii et al., 2008). Briefly, autoclaved bacterial suspension (50 μl) was injected into a silkworm muscle specimen. The contraction value was determined as (prelength-postlength)/prelength. The sample amount (mg) that induced a contraction value of 0.15 was defined as 1 unit.

### Silkworm infection model

The silkworm infection model was described previously (Hamamoto et al., 2004). Pathogens used in the infection model were *P. aeruginosa* PAO1 (Stover et al., 2000) and methicillin-sensitive *S. aureus* 1 (MSSA1) (Akimitsu et al., 1999) from our laboratory stock. Pathogenic bacteria grown in LB medium overnight were diluted with saline (0.9% NaCl) and injected into fifth instar larva (*n* = 7) fed overnight. Survival of silkworm larva was counted for 5 days.

### Statistical analysis

Statistical analysis was performed with Microsoft Excel 2007 for Windows (Redmond, WA, USA) and Excel Statistics 2008 (Social Survey Research Information, Tokyo, Japan). Survival plots were generated by the Kaplan-Meier method and analyzed by the log-rank test. Activity was compared with the Mann-Whitney *U*-test. Differences having a *P* < 0.05 were considered to be statistically significant. Survival curve was plotted using Kaleidagraph 4.1.4 (Synergy Software, Reading, PA, USA) and LD_50_ values were determined from fitting curve of the logistic equation, y = a + (b − a)/(1 + (x/c)^∧^d), y is the fraction of larva killed, x is the number of viable cells injected, c is LD_50_, a, b and d is the constant of fitting curve. Curve fitting was applied with Levenberg-Marquardt algolism.

## Results

### Isolation and characterization of LAB

We selected Gram-positive bacteria on MRS agar, as LAB generally recognized as safe are Gram-positive bacteria. Colonies were re-streaked on CaCO_3_-MRS medium to confirm lactic-acid fermentation. Lactate-fermenting and Gram-positive bacteria were subjected to the silkworm contraction assay. LAB tested for the contraction assay was sequenced for 16S rDNA (Table 1).

**Table 1:** **Identification of bacteria and its activity of silkworm muscle contraction assay**.

**Strain**	**Origin**	**Gram stain**	**Identification (References)**	**GenBank Accession no**.	**ID %**	**Activity (U/mg)**
4	Rice bran pickles	Gram-positive Bacilli	*Lactobacillus sakei* 23K (Chaillou et al., 2005)	NR_075042.1	99	6.7
11-1	Rice bran pickles	Gram-positive Bacilli	*Lactobacillus paraplantarum* DSM10667 (Bringel et al., 1996; Curk et al., 1996)	NR_025447.1	98	165 ± 35^a^
11-2	Rice bran pickles	Gram-positive Cocci	*Pediococcus ethanolidurans* Z-9 (Liu et al., 2006)	NR_043291.1	98	2.7
A	Kimchi	Gram-positive Cocci	*Leuconostoc citreum* KM20 (Kim et al., 2008)	NR_074694.1	100	43

a *Mean ± SE (n = 2)*.

**Figure 1:**
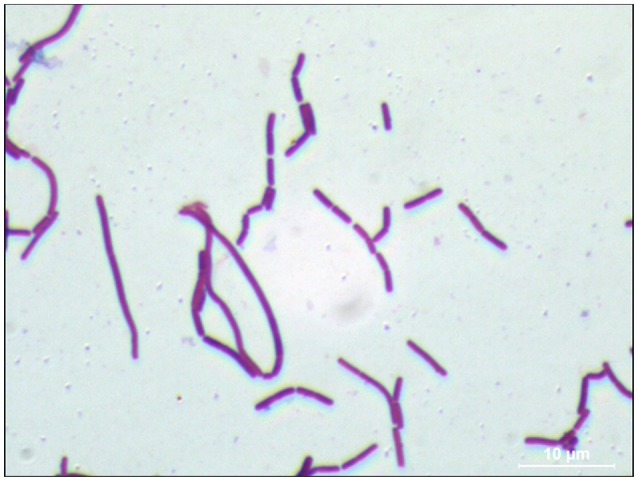
**Gram staining of ***L. paraplantarum*** 11-1**. An *L. paraplantarum* 11-1 colony on CaCO_3_-MRS agar was Gram-stained (Merck). The image was captured with a charge-coupled device camera (Hamamatsu Photonics) using an Olympus phase-contrast microscope at 1,000× magnification.

**Figure 2:**
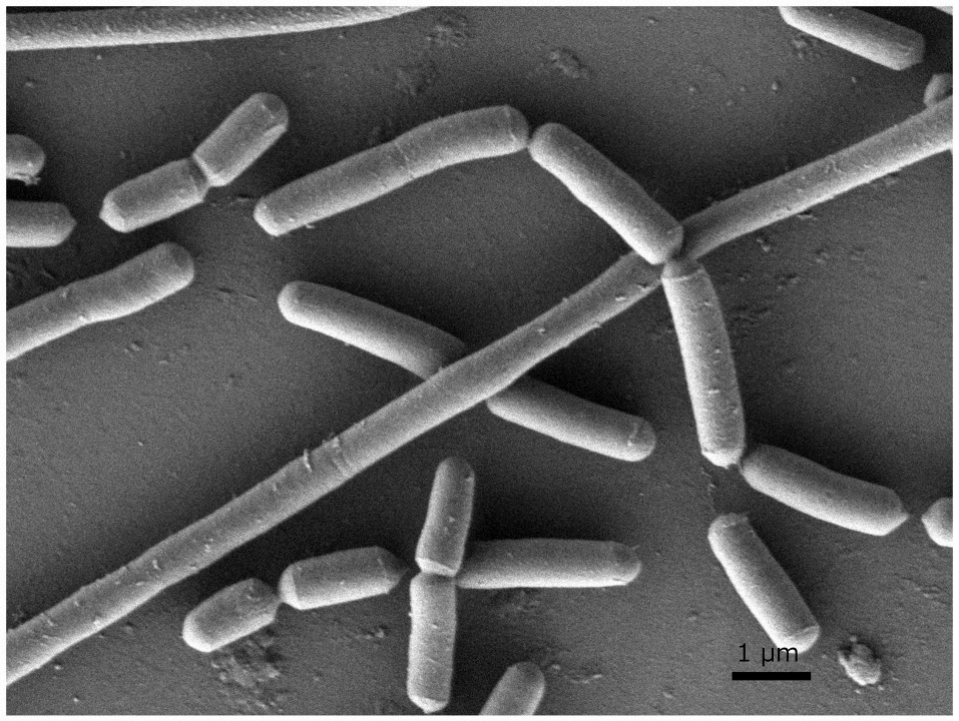
**Scanning electron micrograph (SEM) of ***L. paraplantarum*** 11-1**. The sample was examined with a field-emission SEM (JSM- 7500F, JEOL, Japan). The sample was depicted at 10,000× magnification.

In order to investigate morphological and ultrastructural appearance of isolated bacteria, we performed Gram staining and SEM. Gram stains of LAB displayed a Gram-positive bacillus with homogeneous morphology (Figure 1). SEM revealed that LAB was characterized as a rod shaped bacterium (Figure 2).

The silkworm muscle contraction activity of LAB is shown in Table 1. LAB isolated from pickles showed diverse activity. *L. paraplantarum* 11-1 exhibited the highest activity, 165 U/mg, which was higher than that of the LAB in a previous report from our laboratory (Nishida et al., 2016). The activity of the other isolated LABs was as follows: *Lactobacillus sakei* 4, 6.7 U/mg; *Pediococcus ethanolidurans* 11-2, 2.7 U/mg; and *Leuconostoc citreum* A, 43 U/mg.

BLAST analysis of the 16S rDNA sequence of the 11-1 strain revealed that the 11-1 strain had 98% homology with *L. paraplantarum* DSM10667 (NR_025447.1) and *L. plantarum* WCFS1 (NR_075041.1). Strain 11-1 was more similar to *L. paraplantarum* DSM10667 than *L. plantarum* WCFS1. Therefore, we identified the 11-1 strain as *L. paraplantarum*. We then determined the growth characteristics of *L. paraplantarum* 11-1. *L. paraplantarum* 11-1 had strict temperature sensitivity as it grew in MRS medium at 30°C, but not at 16° or 37°C (Table 2). Growth of *L. paraplantarum* 11-1 in MRS medium was sensitive to salt higher than 5% NaCl in the medium and resistant to acidic conditions (pH 4.0). In contrast, *L. plantarum* JCM1057 grew at a wide-range of temperatures (16°, 30°, and 37°C) and its growth was resistant to salt (5, 8, and 10% NaCl).

**Table 2:** **Growth of LAB in MRS under different conditions**.

**Growth in MRS**	***L. paraplantarum* 11-1**	***L. plantarum* JCM 1057**
16°C	−	+
30°C	+	+
37°C	−	+
43°C	−	−
pH 4, 30°C	+	+
pH 6, 30°C	+	+
pH 7, 30°C	+	+
5% NaCl	−	+
8% NaCl	−	+
10% NaCl	−	+

We next examined the ability of *L. paraplantarum* 11-1 to ferment carbohydrates using Api 50 CHL (Table 3). *L. paraplantarum* 11-1 exhibited different characteristics from *L. paraplantarum* DSM10667 (CNRZ 1885^T^) in 5 of 49 sugars and derivatives utilized. Amygdalin, lactose, melibiose, melezitose, and gluconate were not utilized by *L. paraplantarum* 11-1, whereas α-methyl-D-glucoside was utilized. On the other hand, *L. plantarum* JCM1057 utilized three different sugars than plant-derived *L. plantarum* ATCC14917^T^ (Bringel et al., 1996; Curk et al., 1996). The carbohydrate fermentation scores of *L. paraplantarum* 11-1 matched 90.5% of those of *Carnobacterium maltaromaticum* in the Api web v5.1 database, whereas those of *L. plantarum* JCM1057 matched 99.2% of those of *L. plantarum* 1. The possibility that strain 11-1 was *C. maltaromaticum* was excluded due to the low similarity of the 16S rDNA sequences. The 16S rDNA sequence of strain 11-1 exhibited 98% similarity with *L. paraplantarum* DSM10667 (NR_025447.1) and 91% similarity with *C. maltaromaticum* DSM 20342 (NR_044710.2).

**Table 3:** **Growth characteristics of LAB**.

**Carbohydrate**	**11-1 (This study)**	***L. plantarum* JCM 1057 (This study)**	***L. paraplantarum* DSM 10667^T^ (CNRZ 1885^T^)^a^**	***L. plantarum* subsp. *plantarum* ATCC 14917^T^a^^**
Glycerol	−	−	−	−
Erythritol	−	−	−	−
D-Arabinose	−	−	−	−
L-Arabinose	−	+	−	+
Ribose	±	+	±	+
D-Xylose	−	−	−	−
L-Xylose	−	−	−	−
Adonitol	−	−	−	−
β-Methyl-D-xyloside	−	−	−	−
Galactose	+	+	+	+
D-Glucose	+	+	+	+
D-Fructose	+	+	+	+
D-Mannose	+	+	+	+
L-Sorbose	−	−	−	−
Rhamnose	−	±	−	−
Dulcitol	−	−	−	−
Inositol	−	−	−	−
Mannitol	+	+	+	+
Sorbitol	−	+	−	+
α-Methyl-D-mannoside	−	+	−	+
α-Methyl-D-glucoside	+	+	−	−
N-Acetyl glucosamine	+	+	+	+
Amygdalin	−	+	+	+
Arbutin	+	+	+	+
Esculin	+	+	+	+
Salicin	+	+	+	+
Cellobiose	+	+	+	+
Maltose	+	+	+	+
Lactose	−	+	+	+
Melibiose	−	+	+	+
Saccharose	+	+	+	+
Trehalose	+	+	+	+
Inulin	−	−	−	−
Melezitose	−	+	+	+
D-Raffinose	−	±	+	+
Starch	−	−	−	−
Glycogen	−	−	−	−
Xylitol	−	−	−	−
β-Gentiobiose	+	+	+	+
D-Turanose	−	+	−	+
D-Lyxose	−	−	−	−
D-Tagatose	−	−	−	−
D-Fucose	−	−	−	−
L-Fucose	−	−	−	−
D-Arabitol	−	±	−	±
L-Arabitol	−	−	−	−
Gluconate	−	+	+	+
2-Keto-gluconate	−	−	−	−
5-Keto-gluconate	−	−	−	−

a *Data from Curk et al. (1996)*.

We also examined enzymatic characteristics of strain 11-1 using the Api Zym test and compared them with those of other related strains (Curk et al., 1996; Oberg et al., 2016) (Table 4). The enzymatic characteristics data of *L. paraplantarum* are unavailable, and therefore we compared the data of strain 11-1 and control strain *L. plantarum* JCM 1057, and previously reported data of *L. plantarum* and *L. curvatus*. *L. paraplantarum* 11-1 exhibited different activities (acid phosphatase) of 19 enzymes compared with *L. curvatus* WSU01. In contrast, *L. paraplantarum* 11-1 exhibited 5 different activities (esterase (C4), β-galactosidase, α-glucosidase, β-glucosidase, β-glucosaminidase) of 19 enzymes compared with *L. plantarum* JCM 1057. The enzymatic characteristics of *L. paraplantarum* 11-1 were more similar to those of *L. curvatus* WSU01 than *L. plantarum* JCM 1057.

**Table 4:** **Enzymatic characteristics of LAB**.

**Enzyme**	**11-1 (This study)**	***L. plantarum* JCM 1057 (This study)**	***L. curvatus* WSU01^a^**	***L. planturum* (*n* = 7)^b^**	***L. curvatus* (*n* = 24)^b^**
Alkaline phosphatase	−	−	−	−	−
Esterase (C4)	−	+	−	−	−
Esterase lipase (C8)	−	−	−	−	−
Lipase (C14)	−	−	−	ND	ND
Leucine aminopeptidase	+	+	+	+	+
Valine aminopeptidase	+	+	+	+	+
Cystine aminopeptidase	−	−	−	−	±
Trypsin	−	−	−	ND	ND
Chymotrypsin	−	−	−	ND	ND
Acid phosphatase	+	+	−	+	±
Phosphohydrolase	+	+	+	±	±
α-Galactosidase	−	−	−	±	−
β-Galactosidase	−	+	−	+	+
β-Glucuronidase	−	−	−	−	−
α-Glucosidase	−	+	−	+	−
β-Glucosidase	−	+	−	+	±
β-Glucosaminidase	−	+	−	+	±
α-Mannosidase	−	−	−	−	−
α-Fucosidase	−	−	−	ND	ND

a,b *Data from Oberg et al. (2016) and Papamanoli et al. (2003) respectively*.

### Probiotic effect of *L. paraplantarum* 11-1

**Figure 3:**
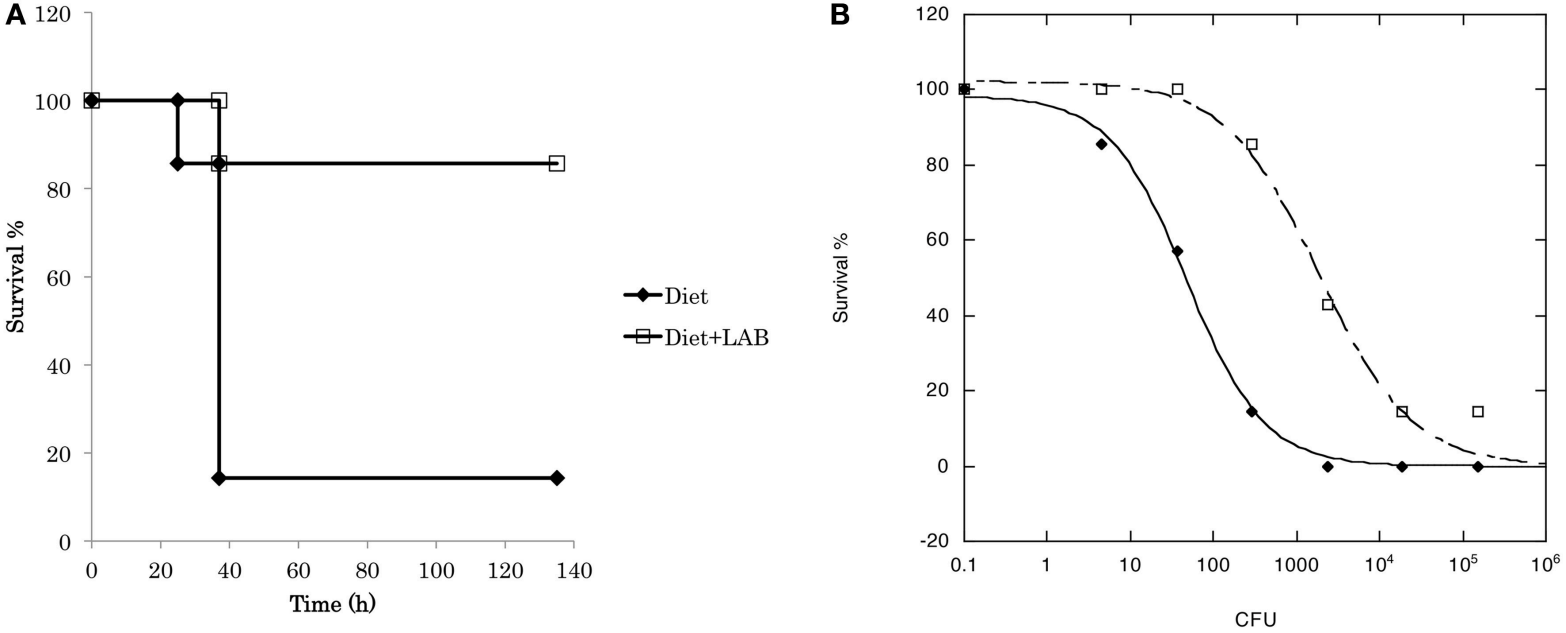
**Probiotic effect of ***L. paraplantarum*** 11-1 on ***P. aeruginosa*** infection. (A)** Time course of survival of silkworms fed a diet with or without *L. paraplantarum* 11-1 viable cells (1 × 10^7^ cfu/larva) after *P. aeruginosa* PAO1 infection. Survival of silkworms fed a diet with *L. paraplantarum* 11-1 was significantly higher than that of silkworms fed a normal diet (*p* = 0.038). **(B)** Dose response of *P. aeruginosa* PAO1 on silkworm survival after 2 days. *P. aeruginosa* PAO1 was injected into 5th instar larva fed a diet with or without *L. paraplantarum* 11-1 viable cells.

**Figure 4:**
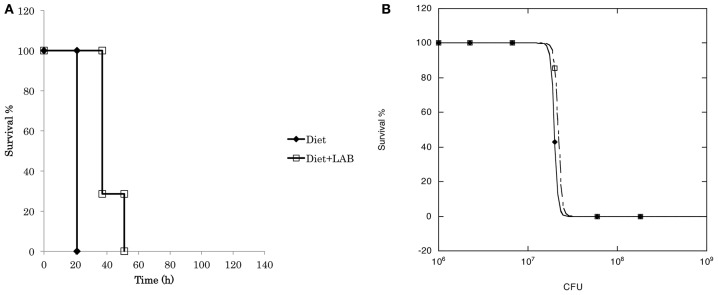
**Probiotic effect of ***L. paraplantarum*** 11-1 on ***S. aureus*** infection. (A)** Time course of survival of silkworms fed a diet with or without *L. paraplantarum* 11-1 viable cells (1 × 10^7^ cfu/larva) after *S. aureus* MSSA1 infection. Survival of silkworms fed a diet with *L. paraplantarum* 11-1 was significantly higher than that of silkworms fed a normal diet (*p* = 0.030). **(B)** Dose response of *S. aureus* MSSA1 on silkworm survival after 2 days. *S. aureus* MSSA1 was injected into 5th instar larva fed a diet with or without *L. paraplantarum* 11-1 viable cells.

To evaluate the probiotic effect of *L. paraplantarum* 11-1, we used the silkworm infection model (Figure 3). Injection of *P. aeruginosa* into silkworm larvae had time-dependent and dose-dependent silkworm killing effects. Silkworms were fed a diet containing *L. paraplantarum* 11-1 viable cells without apparent problems. Feeding silkworms a diet containing *L. paraplantarum* 11-1 viable cells increased the number of animals that survived after injection of *P. aeruginosa*, resulting in a ~100-fold higher LD_50_. We then tested the infection model with the Gram-positive bacteria *S. aureus* (Figure 4). Feeding the silkworm a diet containing *L. paraplantarum* 11-1 viable cells increased the number of animals that survived after injection of MSSA, resulting in a ~2-fold higher LD_50_.

## Discussion

### Isolation of LAB with high innate immunity-stimulating activity

In general, LAB are thought to be beneficial for human health as probiotics in the gut. LAB were recently reported to have high immunity stimulating activity (Ichikawa et al., 2012; Kawashima et al., 2013). Pickles are a food that is fermented with LAB (Hammes, 2009). Fermented-vegetable foods such as pickles are potential sources of non-dairy LAB. In this study, we isolated LAB from pickles by streaking samples of pickle fluid on MRS agar, a selection plate for LAB (de Man et al., 1960). We confirmed the Gram-positive feature of the isolated bacteria and its lactic acid production on CaCO_3_-MRS agar, in which lactic acid solubilizes CaCO_3_ to form a transparent zone around the colony. Gram staining and SEM characterized LAB as a Gram-positive bacillus and a rod shaped bacterium respectively. (Figures 1, 2). Pleomorphism, defined as variation in size or shape of a bacterial cell, is described for different Lactobacillicae in response to the absence of deoxyribosides, vitamin B_12_, or divalent cation. Culture broth composition is correlated with the morphology of *L. acidophilus* NCFM (Senz et al., 2015). Different sizes in microscopy morphology might depend on nutrient composition in culture medium. Next, we determined the 16S rDNA sequences of each isolate. The identity of strain 11-1 as *L. paraplantarum* was based on 98% similarity between strain 11-1 and *L. paraplantarum* DSM10667 (NR_025447.1).

*L. plantarum* and *L. paraplantarum* are closely related and heterofermentive. *L. paraplantarum* is isolated from beer and human feces. *L. plantarum* is a nonpathogenic LAB colonizing in fermented foods and in the human mouth and gut. Therefore, *L. plantarum* is normal human gut microbiota (Bernardeau et al., 2008; Hammes, 2009). The *L. plantarum* WCFS1 genome was sequenced and a recombinant DNA technique was established to construct a strain expressing a specific antigen (Grangette et al., 2001; Seegers, 2002; Kleerebezem et al., 2003; Wells and Mercenier, 2008; Siezen et al., 2012).

### Innate-immunity activation in silkworms

Multiple studies demonstrate the validity of silkworm contraction assay for innate immune activation (Ishii et al., 2008; Dhital et al., 2011; Fujiyuki et al., 2012). We determined the activity of LAB to stimulate innate immunity in silkworms using a muscle contraction assay (Ishii et al., 2008). When *L. paraplantarum* 11-1 was injected into the silkworm body fluid, the insect cytokine paralytic peptide was activated upon innate immune stimulation, resulting in silkworm muscle contraction. Compared with a conventional method using macrophages, the muscle contraction assay does not require cell culture and is insensitive to lipopolysaccharides, which often cause a false-positive response in test samples. We isolated several LABs that exhibited a variety of muscle contraction activities (Nishida et al., 2016). Among them, *L. paraplantarum* 11-1 exhibited the highest activity (Supplemental Figure 1).

### Silkworm acquired tolerance to bacterial infection by ingesting *L. paraplantarum* 11-1

Use of silkworms as a surrogate animal for animal tests poses the fewer ethical, financial, and logistical problem than mammalian tests. In addition, silkworms are large enough to inject a precise amount of samples compared to other insect (Sekimizu et al., 2012). The infection model described here also has the potential to be used to study novel probiotics for *in vivo* activity against *P. aeruginosae*.

We have used the silkworm as a surrogate animal to test the probiotic effect of LAB (Nishida et al., 2016). Silkworms were fed a diet containing LAB. Silkworms fed LAB exhibited tolerance to the lethality of *P. aeruginosa* and *S. aureus* infections. Our previous results demonstrated that silkworms acquire tolerance to *P. aeruginosa* infection by ingesting a diet containing *Lactococcus lactis* or peptidoglycans of *Lactobacillus plantarum* (Miyashita et al., 2015; Nishida et al., 2016). In this study, we demonstrated that ingesting *L. paraplantarum* 11-1 extended the survival of silkworm after infection with *P. aeruginosa*. These findings suggest that activation of the innate immune system induced tolerance against microbial infection.

LAB in dairy and fermented products are expected to benefit human health. Reports on the probiotic effects of LAB in animal infection models, however, are limited. Oral administration of heat-killed *L. casei* protects against *P. aeruginosa* infection in mice (Miake et al., 1985; Setoyama et al., 1985). *Bifidobacterium longum* prevents *P. aeruginosa* gut-derived sepsis in a mouse model (Matsumoto et al., 2008). *Bifidobacterium* protects germ-free mice from *E. coli* O157 infection (Fukuda et al., 2011). Our data indicate that the silkworm is a useful model animal for evaluating the probiotic effects of LAB. *P. aeruginosa* is the most commonly isolated antibiotic-resistant Gram-negative bacteria in ventilator-assisted pneumonia. Oral administration of a probiotic delays respiratory tract colonization and infection by *P. aeruginosa* in human (Forestier et al., 2008). Searching novel probiotics would be matching potential medical needs. Further study on the prevention of *P. aeruginosa* in silkworm infection model would be required for the translation of probiotics to benefit human health.

## Ethics statement

This study was exempted by The University of Tokyo Life Science Research Ethics and Safety Committee, and Teikyo University Animal Ethics Committee.

## Author contributions

SN designed and conducted the experiments, and performed data analysis. MI and YN conducted the experiment of Gram stain and SEM. SN and KS wrote the manuscript. MI, YN, SA, and YO reviewed and edited the manuscript. SN, YO, and KS revised the manuscript.

### Conflict of interest statement

KS is a consultant for Genome Pharmaceuticals Institute Co. Ltd. The other authors declare that the research was conducted in the absence of any commercial or financial relationships that could be construed as a potential conflict of interest.
