# Supplementary material for: Lactobacillus paraplantarum 11-1 Isolated from Rice Bran Pickles Activated Innate Immunity and Improved Survival in a Silkworm Bacterial Infection Model
Source: Front Microbiol. 2017 Mar 20;8:436. doi: 10.3389/fmicb.2017.00436 (PMC5357627; doi:10.3389/fmicb.2017.00436)

**Supplemental Figure 1. Silkworm muscle contraction activity induced with various LAB.** Summary data from Table 1 in this report, and Table 2 in the previous report (Nishida et al. 2016). *P < 0.05 (Mann–Whitney U test).


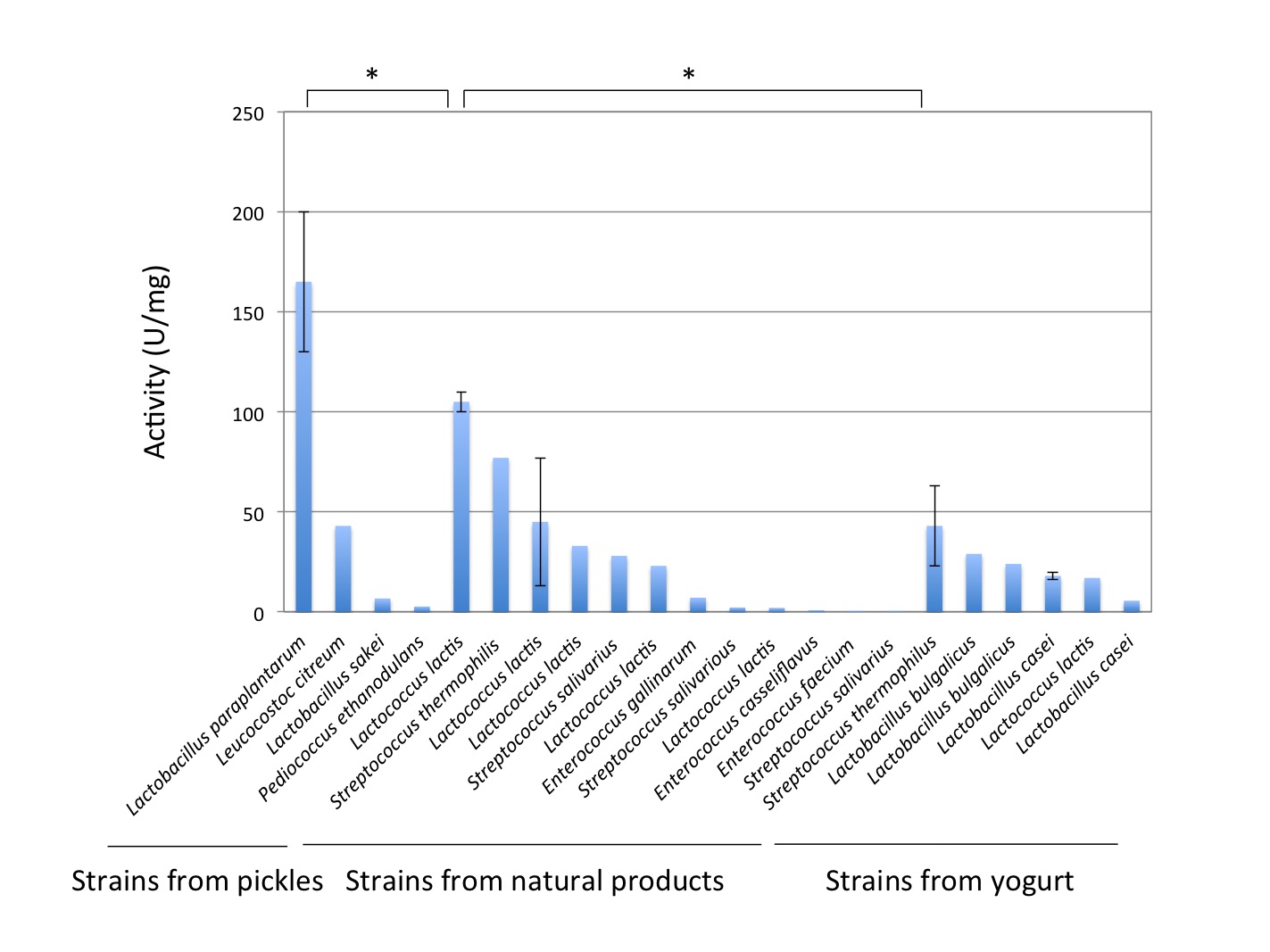

Supplement: Supplementary file 1 [file DataSheet1.docx]
